# Trends in Fetal Growth Between 2000 to 2014 in Singleton Live Births from Israel

**DOI:** 10.1038/s41598-018-19396-w

**Published:** 2018-01-18

**Authors:** Keren Agay-Shay, Mary Rudolf, Lisa Rubin, Ziona Haklai, Itamar Grotto

**Affiliations:** 1 Azrieli Faculty of Medicine, Bar Ilan University, Safed, Israel; 2 Department of Maternal and Child Health, Public Health Services, Ministry of Health, Jerusalem, Israel; 3 School of Public Health, University of Haifa, Haifa, Israel; 4 Health Information Division, Ministry of Health, Jerusalem, Israel; 5 Ministry of Health, Jerusalem, Israel; 6 Faculty of Health Science, Ben-Gurion University of the Negev, Beer Sheva, Israel

## Abstract

Trends in birthweight and abnormal fetal growth, namely term low birthweight (LBW), macrosomia, small-for-gestational age (SGA) and large-for-gestational age (LGA), are important indicators of changes in the health of populations. We performed this epidemiological study to evaluate these trends among 2,039,415 singleton live births from Israel over a period of 15 years. Birth certificate data was obtained from the Ministry of Health. Multivariable linear and logistic regression models were used to evaluate crude and adjusted estimates compared to the baseline of 2000 and polynomial trends. During the study period we observed a significant decrease in the rates of infants born SGA and LGA (10.7% to 9.2%, 10.2% to 9.6% respectively). After adjustment, based on the imputed data set, term mean birthweight increased by 6.0 grams (95% CI: 2.9, 9.1), and term LBW odds decreased by 19% in 2014 compared to 2000 (adj ORs: 0.81; 95% CI: 0.77, 0.85). Significant decreases were also observed for adjusted SGA, LGA and macrosomia rates. The decrease in abnormal fetal growth rates were not entirely explained by changes in sociodemographic characteristics or gestational age and may imply real improvement in child intrauterine growth in Israel during the last 15 years, especially in the Jewish population.

## Introduction

Birthweight is an important indicator of the nutritional and developmental status of a newborn infant^1^. Abnormal fetal growth is commonly identified using criteria such as low birthweight (LBW) and small-for-gestational age (SGA) for the small fetus and macrosomia and large-for-gestational age (LGA) for the large fetus^2,3^. Studies show that both LBW and macrosomia are related to adverse short-term and long-term health outcomes. Macrosomia is an important risk factor for perinatal asphyxia, death, and shoulder dystocia^4^. LBW is strongly associated with perinatal mortality and child morbidity^5,6^. The associations between LBW and macrosomia with adverse health outcomes have been reported over the full life course^1,7^. Research has shown that LBW is associated with increased coronary heart disease, high blood pressure and type 2 diabetes and other chronic diseases later in life^8,9^. Abnormal weight gain in the uterus and during infancy may have an adverse influence on health in childhood and adult life and children with LBW and macrosomia tend to gain weight faster than those born at normal weight^10,11^. These highlight the importance of studying both low birthweight and high birthweight as adverse pregnancy outcomes. Since the beginning of the millennium many registry-based studies around the world have analyzed time trends in normal and abnormal fetal growth as an important indicator for public health (summarized in Table S1^12–38^) however, only a few of these studies evaluated both small and large abnormal fetal growth^13,15–17,19–34,36,38^.

Israel is a unique country with the highest fertility rate among the OECD countries. Despite high levels of maternal education and access to modern contraception, the total rate of 3.1 births per woman in 2015 was more than 30% greater than for women in the next-ranked country in the OECD^39^. A previous Israeli hospital-based study of 32,611 births, compared rates during 2003–2004 and 1994–1996 to 1986–1987 rates and reported crude decrease in the rates of abnormal fetal growth on both sides of the birthweight distribution^40^.

In this population based epidemiological study our objectives were: 1) to evaluate time trends of normal and abnormal fetal growth from both sides of the distribution, in 2,039,415 singleton live births from Israel over a period of 15 years (from 2000 until 2014) and 2) to evaluate if these trends might be explained by changes in child and maternal sociodemographic characteristics.

## Results

### Descriptive statistics

**Figure 1:**
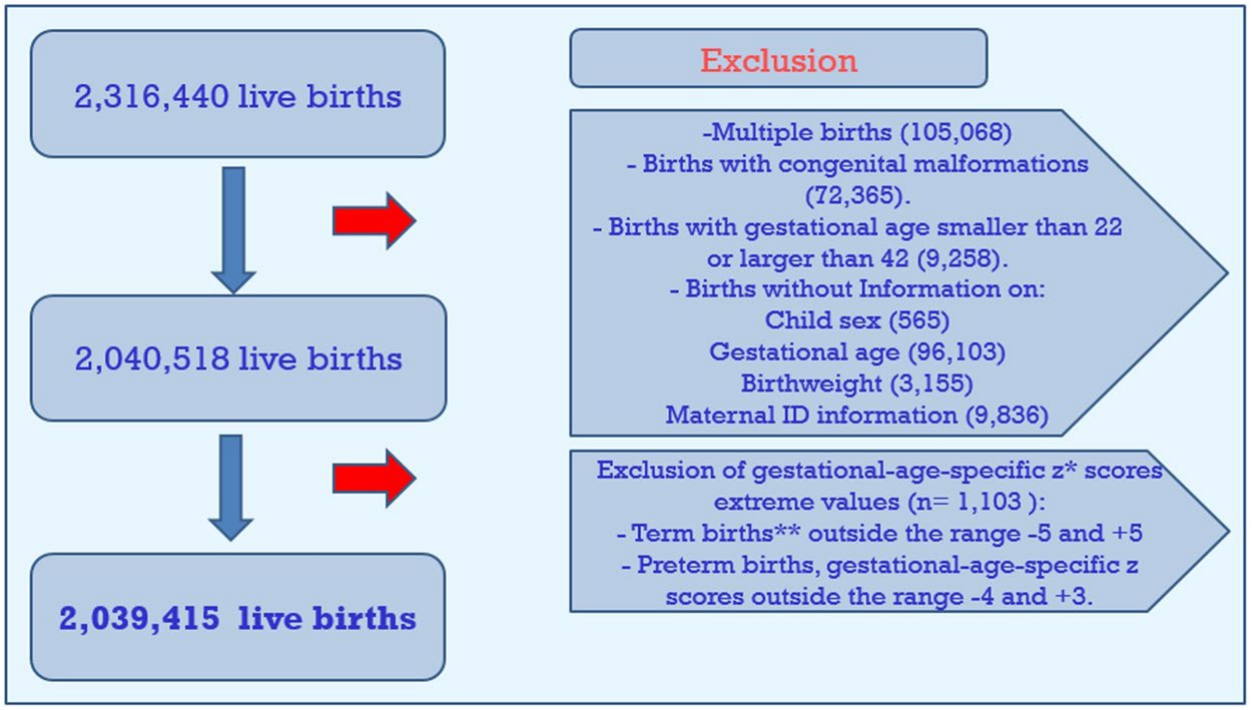
Research population. *z scores = (observed birthweight -mean birthweight)/standard deviations, where the mean and the standard deviations are based on sex-specific births at the observed gestational age (in completed weeks), based on our study population. **Term births-gestational age equal or above 37 weeks.

During the period studied, there were 2,039,415 singleton live births born at 22–42 weeks gestation without congenital malformations, including normal and abnormal fetal growth (Fig. 1). The mean and standard deviation of birthweight in total and for term births was 3254 ± 494 gr and 3304 ± 435 gr, respectively. The rates of term LBW, SGA, LGA, macrosomia >4000 gram and macrosomia >4500 gram were: 2.7%, 9.8%, 9.8%, 5.4% and 0.5% respectively.

Changes from 2000 to 2014 are presented in Table 1. Mean and standard deviation of birthweight for total births decreased from 3268 ± 509 gr during 2000 to 3255 ± 484 gr during 2014. The decrease was consistent until 2008 and then fluctuated until 2014. A similar pattern was observed for the term birth population. The trend of decrease in the rates of macrosomia (>4000 gr and >4500 gr) followed the pattern of birthweight. The rates of macrosomia decreased steadily from 2000 until 2008 and then fluctuated until 2014(6.04% in 2000 to 5.14% in 2014; 0.59% in 2000 to 0.40% in 2014; respectively). Although mean birthweight in term deliveries decreased, the rate of term LBW infants was fairly stable with a small decrease from 2.84% in 2000 to 2.59% in 2014. The decrease in the rates of SGA infants (from 10.72% in 2000 to 9.16% in 2014) was larger than the decrease in the rates of LGA infants (from 10.22% in 2000 to 9.55% in 2014).

**Table 1:** Characteristics (mean or percent) of singleton births by year, 2000–2014, Israel.

	2000	2001	2002	2003	2004	2005	2006	2007	2008	2009	2010	2011	2012	2013	2014
**Total births(n)**	116285	117632	120906	123813	124425	124579	128809	133064	139059	144575	146987	150561	153331	155310	160079
**Gestational age (weeks); mean(SD)**	39.34; (1.82)	39.28; (1.79)	39.25; (1.80)	39.23; (1.79)	39.18; (1.80)	39.14; (1.76)	39.13; (1.72)	39.11; (1.73)	39.12; (1.73)	39.11; (1.72)	39.14; (1.75)	39.15; (1.73)	39.16; (1.71)	39.13; (1.69)	39.11; (1.68)
**Birth weight (gram); mean(SD)**	3267.93; (509.07)	3267.43; (504.40)	3263.83; (502.73)	3258.93; (498.53)	3257.06; (498.14)	3255.56; (498.60)	3253.58; (492.94)	3246.17; (492.11)	3244.86; (491.43)	3246.48; (492.17)	3250.31; (490.79)	3248.12; (488.21)	3252.11; (488.17)	3253.88; (484.28)	3255.47; (484.25)
**LBW (%)**	5.50	5.47	5.45	5.44	5.52	5.58	5.45	5.57	5.51	5.44	5.36	5.33	5.34	5.16	5.15
**Macrosomia 4000 gr (%)**	6.04	5.98	5.80	5.56	5.46	5.43	5.37	5.13	5.00	5.16	5.22	5.12	5.21	5.12	5.15
**Macrosomia 4500 gr(%)**	0.59	0.56	0.52	0.53	0.51	0.46	0.45	0.42	0.41	0.42	0.41	0.42	0.41	0.42	0.40
**AGA (%)**	79.06	79.48	79.90	79.94	80.02	80.37	80.16	80.47	80.85	80.77	80.87	80.91	80.87	81.30	81.29
**SGA (%)**	10.72	10.28	9.96	10.11	9.83	9.55	9.77	9.79	9.75	9.62	9.58	9.70	9.70	9.24	9.16
**LGA (%)**	10.22	10.23	10.13	9.95	10.16	10.08	10.07	9.74	9.40	9.61	9.55	9.40	9.43	9.46	9.55
**Term births(n)**	110057	111350	114166	116825	116880	117500	121679	125486	131438	136782	139034	142740	145580	147511	152047
**Term LBW (%)**	2.84	2.81	2.68	2.75	2.80	2.86	2.80	2.81	2.75	2.70	2.66	2.71	2.74	2.63	2.59
**Term Gestational age(weeks); mean(SD)**	39.63; (1.24)	39.56; (1.23)	39.55; (1.23)	39.52; (1.22)	39.49; (1.22)	39.42; (1.22)	39.41; (1.20)	39.38; (1.21)	39.39; (1.21)	39.38; (1.21)	39.41; (1.23)	39.41; (1.23)	39.41; (1.23)	39.38; (1.22)	39.36; (1.21)
**Term birth weight (gram); mean(SD)**	3318.66; (445.33)	3317.65; (442.91)	3315.67; (439.48)	3309.57; (437.27)	3308.81; (438.46)	3306.79; (438.17)	3302.86; (435.46)	3297.07; (433.4)	3295.50; (430.83)	3296.59; (431.96)	3300.41; (430.88)	3296.11; (430.76)	3299.44; (432.14)	3300.54; (428.81)	3302.41; (428.48)

AGA-appropriate gestational age (10^th^ to 90^th^ percentile); LBW- Low birthweight; LGA-large for gestational age by sex (above 90^th^ percentile); SGA- small for gestational age by sex (under 10^th^ percentile); SD-standard deviation; Term births-births at gestational age equal or above 37 weeks.

**Figure 2:**
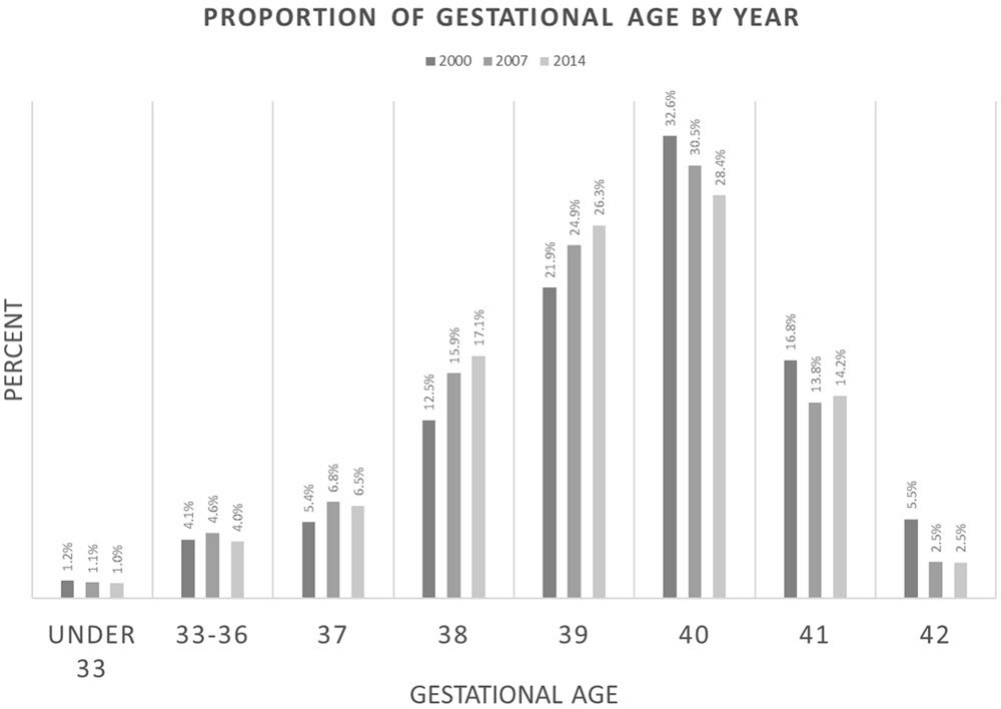
Singleton births by gestational age category: 2000, 2007, and 2014.

Changes in rates of births by gestational weeks are shown in Fig. 2 and Supplemental Material Table S2. The rates of births at gestational age of 37–39 weeks increased and rates of births with gestational age of 22–36 and 40–42 weeks decreased. Changes in the proportion of the sociodemographic characteristic categories from 2000 to 2014 are presented in Fig. 1S.

### Trends in mean birthweight and abnormal fetal growth

**Figure 3:**
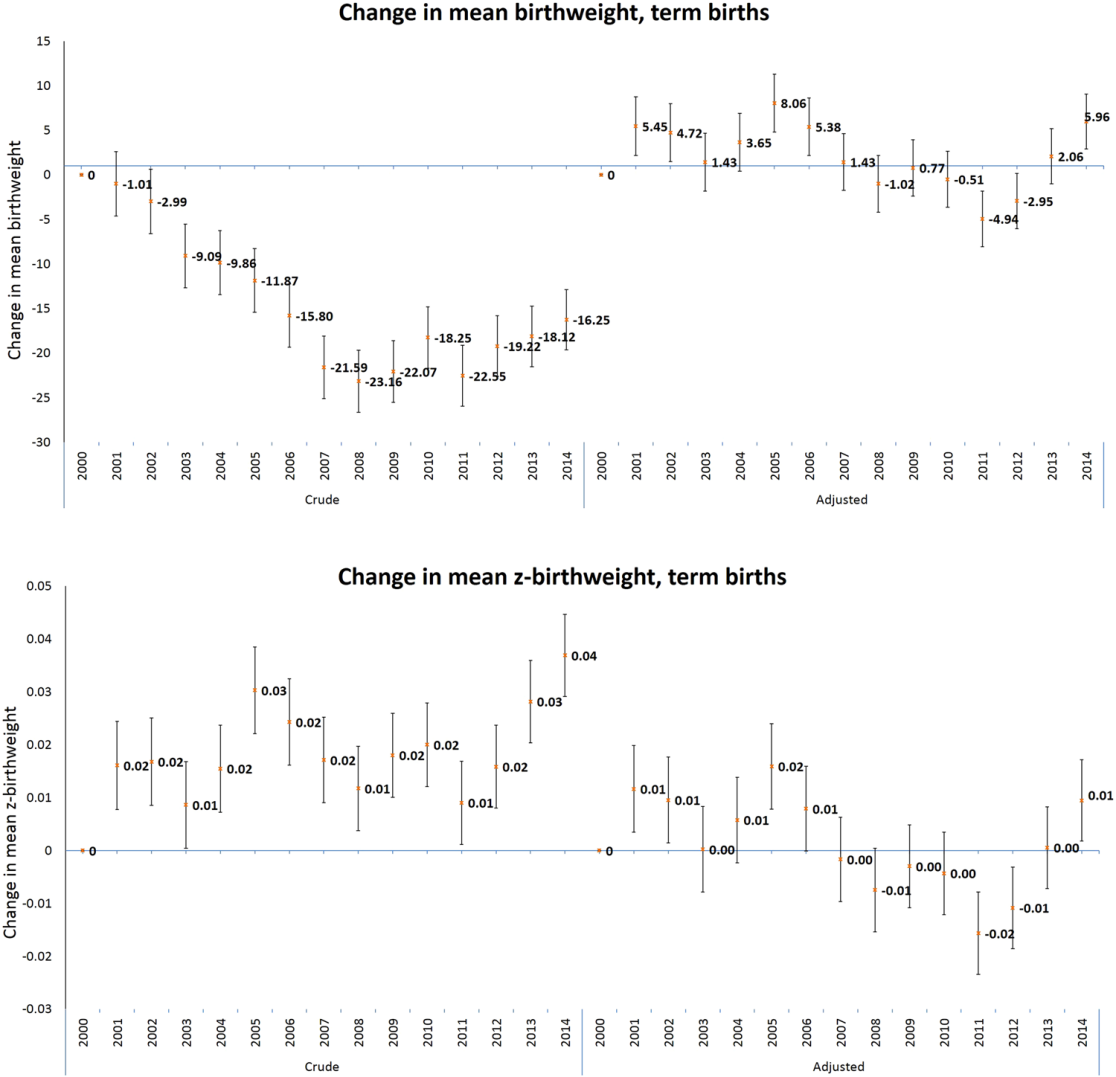
Crude and adjusted * change in mean birthweight and z-birth weight for term births** and 95% confidence intervals (95% CI) compared to the baseline of year 2000, by year of birth, imputed data. *Adjusted to gestational age, child sex, child religion, season of conception, maternal parity, maternal origin of birth, maternal family status, maternal age and maternal education. **Term births-gestational age equal or above 37 weeks.

**Figure 4:**
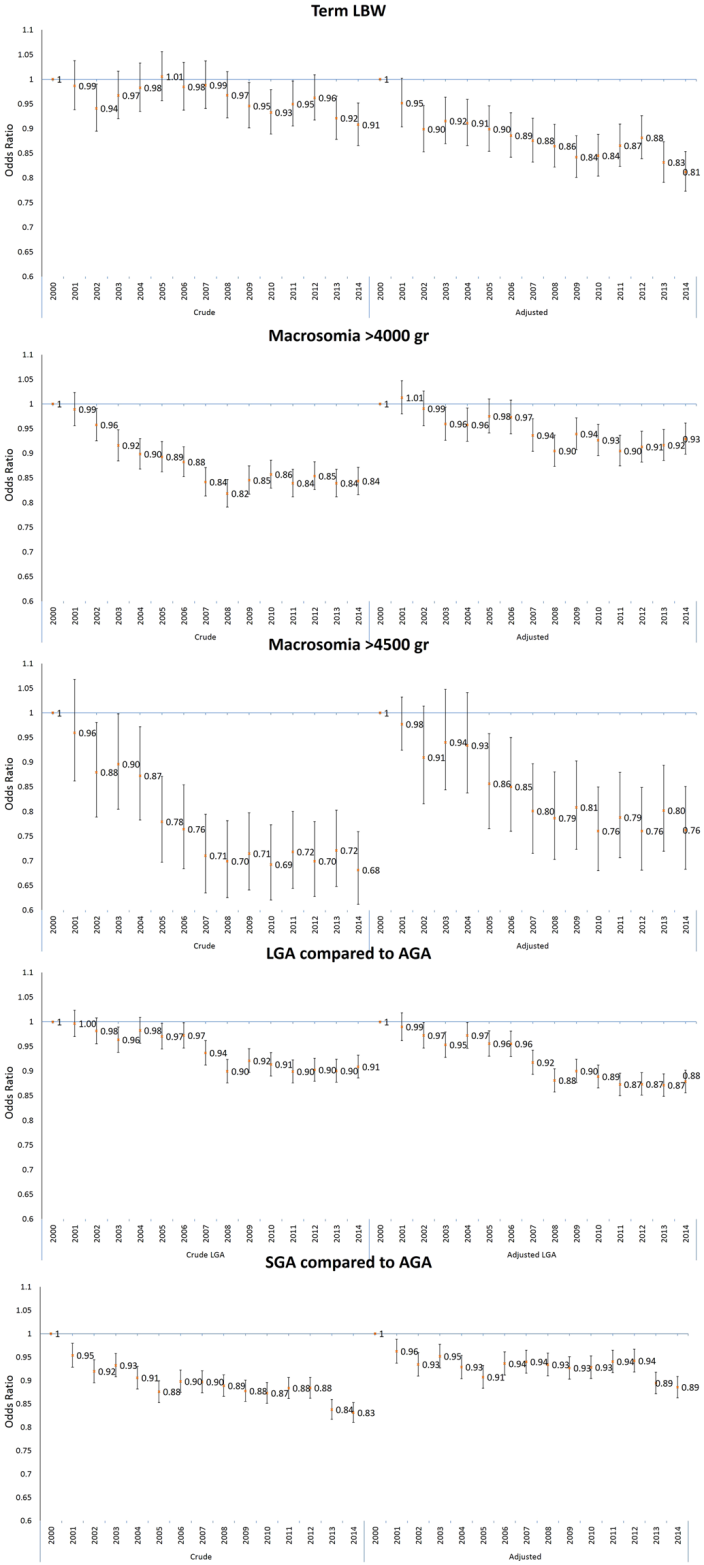
Crude and adjusted* ORs(Odds Ratio) and 95% Confidence intervals(95% CI) for term LBW, macrosomic births and SGA and LGA compared to AGA, by year of birth, compared to the baseline of year 2000, imputed data. GA-Appropriate gestational age(10^th^ to 90^th^ percentile); LGA-large for gestational age by sex(>90^th^ percentile); macrosomic births (>4000 gr and >4500 gr); Term LBW- Low birthweight born at gestational age equal or above 37 weeks; SGA- small for gestational age by sex (<10^th^ percentile). *All models were adjusted to gestational age, child sex, child religion, season of conception, maternal parity, maternal origin of birth, maternal family status, maternal age and maternal education.

Analysis of the crude and adjusted (based on the imputed data set) changes in mean birthweight, z-birthweight and changes in abnormal fetal growth are presented in Figs 3 and 4 (and Supplemental Material Tables 3S and 4S). Crude mean birthweight in term deliveries decreased by 16.25 gram in 2014 compared to 2000 (95% CI (Confidence Interval): 12.9, 19.6). The greatest decrease in estimated OR (Odds Ratio) in the crude models were observed for macrosomia (>4500 gr). The crude OR for macrosomia (>4500 gr) in 2014 compared to 2000 was 0.68 (95% CI: 0.61, 0.76).

Complete-case estimates, based on the adjustment to all covariates (except for maternal education) (Supplemental Material Table 5S), demonstrated stronger trends compared to the adjusted estimates based on the imputed data set and estimates based on models with “unknown” category (Supplemental Material Table 6S). Generally, adjusted estimates based on the imputed data set were similar to estimates based on models with “unknown” category. Adjustment for all the covariates, based on the imputed data set, had a generally small effect on the point estimates of the temporal analysis for LGA and SGA compared to the crude models. The adjusted ORs for SGA in 2014 compared to 2000 was 0.89 (95% CI: 0.86, 0.91), and for LGA a similar decrease in 2014 compared to 2000 was observed (adj ORs: 0.88 95% CI: 0.86, 0.90). However, for term births mean weight and mean z-birthweight, term LBW and for macrosomia (>4000 gr and >4500 gr) notable changes were observed between the crude and adjusted estimates. The estimated associations after adjustment changed from decrease to an increase in term births mean weight in 2014 compared to 2000 (adj β: 5.96; 95% CI: 2.88, 9.05) and completely attenuated the small effects observed for mean z-birthweight in the crude models. After adjustment, the increase in mean birthweight also changed the ORs of term LBW and the ORs in 2014 compared to 2000 demonstrated a stronger decrease (crude ORs: 0.91; 95% CI: 0.87,0.95 and adj ORs: 0.81; 95% CI: 0.77, 0.85). Surprisingly, the increase in mean birthweight did not increase the risk for macrosomia; however, adjustment did attenuate the decrease that was observed in the crude models. The adjusted ORs for macrosomia (>4000 gr and >4500 gr) in 2014 compared to 2000 was 0.96 (95% CI: 0.93, 0.99) and 0.77 (95% CI: 0.70, 0.86), respectively.

Polynomial trend analysis of the crude and adjusted changes in mean birthweight, z-birthweight and in abnormal fetal growth are presented in Supplemental Material Tables 3S, 4S, 5S and 6S. Polynomial trend analysis based on the imputed data set demonstrated for the adjusted models linear and non-linear significant decrease trends for term LBW, SGA, LGA and a significant polynomial increase trend for birthweight and z-birthweight (Supplemental Material Table 4S).

All the changes in the adjusted compared to the crude estimates were mainly attributable to the adjustment for gestational age and maternal education. Additional adjustment for infant’s sex, infant’s religion, parity, maternal age, maternal marital status, maternal origin and season of conception had minimal influence on the estimates.

There were no statistically significant differences regarding the changes over time in fetal growth between maternal education categories (high - postsecondary and low/medium - secondary or less) however, statistically significant differences between the infants’ religion categories (Jewish and Muslim) were observed. (Supplemental Material Fig. 2S for changes in mean birth weight, results for term LBW, macrosomia, SGA and LGA and z mean birth weight, are not presented).

## Discussion

Our study demonstrated a decrease in the crude mean and standard deviations of birthweight reflecting a decrease in the distribution of birthweight during 15 years. We also observed a decrease in the crude rates of abnormal fetal growth, namely macrosomia, SGA and LGA births. In the Israeli population, changes occurred over the study period for gestational length and in various sociodemographic characteristics. After adjustment, results for SGA and LGA did not change, mean birthweight increased; and term mean z-birthweight, term LBW and macrosomia attenuated. The changes in the adjusted compared to the crude associations were mainly attributable to the adjustment for gestational age and maternal education. The stratified analysis by child religion revealed that the changes occurred mainly in the Jewish population.

Many studies based on registry birth data have evaluated trends in normal and abnormal fetal growth. Previous studies have either demonstrated a trend of decrease in crude mean birthweight (and increase in the proportion of small babies)^12–16,19–33,36,41,42^, or demonstrated an increase in crude mean birthweight (and an increase in the proportion of large babies)^17,18,38^. To our knowledge previous studies evaluating both small and large abnormal fetal growth distribution reported improvement only in one side of the distribution^13,15–17,19–34,36,38^. In our study we observed on a population level, a temporal improvement in abnormal fetal growth on both sides of the weight distribution, i.e for large and small infants.

The change observed in our study in mean birthweight, after adjustment, was quite small (an increase of 5.96 g) and might not have clinical importance at an individual level. Our result after adjustment of increase in mean birthweight are consistent with studies from China^17^, Wales^38^ and Denmark^18^ but inconsistent with studies from the USA^13,15^ and France^36^.

A notable benefit at the population level was observed; after adjustment the odds of term LBW births decreased by 19% and the odds of macrosomia (>4000 gr and >4500 gr) births decreased by 7% and 24% respectively in 2014 compared to 2000. Previous studies have shown that both LBW and macrosomia are related to adverse health outcomes throughout the life course^1,7^ as explained by the ‘Barker theory’ and the ‘rapid catch-up growth hypothesis’^8,9^. According to Barker’s theory, birthweight is a marker of newborn health, reflects intrauterine fetal growth and is a sentinel for health outcomes later in life^8^. Abnormal weight gain during infancy may have an adverse influence on health in childhood and adult life^10,11^. Children born LBW often show “catch-up” growth, and rapid weight gain at this age and macrosomia are associated with later obesity. Therefore, the observed decrease in abnormal fetal growth rates may be a positive indicator for Israeli population health.

Israel is a unique country with the highest fertility rates among the OECD countries despite the high levels of maternal education and access to modern contraception. Even non-religion Jewish women, who have the lowest fertility rates among Israeli women, have higher fertility rates than women in any other OECD country. The overall general fertility rate is slowly rising and was 3.05 in 2013, and reflects a slow rise in the fertility rate of the Jewish population, which is offset by a 25% decline in the general fertility of the Muslim population from 4.7 to 3.35 over the last 13 years^43^. In Israel, the leading cause of infant mortality among Jews was preterm birth and other perinatal disorders and for Arab (Muslim, Christian and Druze), congenital malformations and inherited diseases. The observed temporal improvement in abnormal fetal growth rates may partly explain the recent observation of temporal decrease in infant mortality rates in the Israeli population^39^. In our study we did not observe differences in the trends among the education categories tested. However, we observed differences in the trends between the religion categories tested. This could be partly explained by the differences in the trends of smoking between Jewish and Muslim women. A recent report described a decrease trend in smoking rates among Jewish women (25% during 2000 to 15% during 2015) but not for Muslim women (5.8% during 2000 to 6.7% during 2015). For other characteristics such as delay in childbearing and an increasing education level trend were generally similar between Jewish and Muslim women^39,43–45^.

The observed trends in abnormal fetal growth might be attributable to changes in obstetric practice, such as earlier delivery of large neonates, reducing post term births and better supervision and treatment of pregnancies at risk with a reduction in preterm and very preterm deliveries. These practices have clear implications for improvement in the Israeli health care system, as described elsewhere^43^. In Israel caesarean birth rates are the lowest among OECD countries (20%). Caesarean rates at all live births (singleton and multiple births) have increased since 2000 to 2006 (14% to 16.4%), and leveled off to 16.2% during 2015^46^. Although data on trends of Caesarean rates at singleton births and other obstetric practice was not available, we observed changes in gestational length, and a decrease in preterm and post-term deliveries. Our results for fetal growth were stable after the adjustment for gestational length. We also saw a decrease in the rates of SGA and LGA births, which may imply a real improvement in neonatal growth patterns^47^. SGA is of course not a synonym for intrauterine growth restriction. It is a statistical definition, and includes small but otherwise healthy infants as well as infants who have not achieved their growth potential. By contrast, intrauterine growth restriction is a condition where the fetus is unable to achieve its growth potential and is assessed by longitudinal evaluation of fetal growth rate by ultrasonography during pregnancy. So saying, SGA may be regarded as a proxy of intrauterine growth restriction^48^, so the decrease in SGA births observed in our study may reflect a reduction in intrauterine growth restriction in the Israeli population. Similarly, the reduction in LGA births may imply an improvement in treatment of conditions such as gestational diabetes that cause macrosomia^49^. Further studies are needed to test these hypotheses.

A limitation of our study was the lack of information available on the Israeli birth certificate such as maternal BMI and weight gain, pathologies (such as preeclampsia and gestational diabetes), illness, alcohol, tobacco and drug use during pregnancy, all of which affect fetal growth^50^. So saying, adjustment for maternal education and age, which are associated with these factors, did not change significantly the estimates observed. Other limitations are inherent to birth record data, but birthweight tended to be well recorded and most of the sociodemographic factors are based on data collected and well documented by the civil registry (MIRSHAM OCHLOSIN). The dataset included estimates of gestational age based on ultrasound measures and calculation by date of last menstrual period. The percentage of missing data in this field was negligible and did not change with time and so misclassification in the determination of gestational age was unlikely to be biased by the yearly trends. Gofin *et al*. (2004)^51^ who conducted a survey among a representative sample of urban areas of 1,100 Israeli pregnant women who gave birth in March 2000 showed that nearly all of the women had an ultrasound scan during pregnancy (98% of Jewish women and 95% of Arab women). Years of education, religiosity, age, number of children, and pathology of pregnancy were not associated with the performance and frequency of ultrasound scans among Jewish and Arab women. Thus an already high uptake of ultrasound examinations was found from the beginning of the study period. We could not find information regarding trends in ultrasound performance in the general population however we have no information that ultrasound use has declined and the Ministry of Health mandated four scans in 2013, updating previous recommendations for two scans. We assume that trends in ultrasound performance could cause inaccuracies in gestational age determinations and will cause misclassification, however, the misclassification is assumed to be random (no differences between pregnant women with adverse fetal growth outcomes or without adverse fetal growth outcomes) and, therefore, is most likely to bias results towards the null.

The strength of our study is the use of a national registry-based data set that included all births in Israel over a period of 15 years. This provided a large population, and allowed examination of small yet consistent changes in abnormal fetal growth over time. Unlike smaller and hospital-based studies^40^, our study permitted us to generalize the findings to the entire population of Israel.

To conclude, in this study we observed a decrease in abnormal fetal growth in 2,039,415 Israeli singleton live births over 15 years. This could not be explained by changes in maternal and neonatal socio-demographic characteristics, and the changes in gestational age also did not entirely explain this decline. Our findings may reflect changes in obstetric practice and may also indicate real improvement in child intrauterine growth.

## Methods

### Population

Birth certificate data on all live births from 2000 to 2014 were obtained from the National Birth and Birth Defect Registry, which is operated by the Department of Mother and Child Health in the Public Health Service of the Israel Ministry of Health (N = 2,316,440.) Reporting of all live births is obligatory to the Ministry of Interior and to the Ministry of Health by Israeli law. Reports include infant birth outcomes and parental sociodemographic characteristics, namely years of maternal education, religion, maternal marital status and origin.

We excluded multiple births (N = 105,068) and births without information on maternal ID (N = 9,836), child sex (N = 565), gestational age (N = 96,103), birthweight (N = 3,155) or births with congenital malformations (N = 72,365). We excluded births with gestational age below 22 or above 42 weeks (N = 9,258). Population size after exclusion was 2,040,518 births. (see Fig. 1).

### Correction of birthweight for sex and gestational age

Birthweight was reported in 10 gram units. Gestational age was reported in completed weeks, based on reported date of the last menstrual period (LMP) in conjunction with confirmatory ultrasound or in the case of discrepancy between LMP and ultrasound estimation, by ultrasound performed up to 20 weeks. Israeli women are entitled to four ultrasound examinations during pregnancy. Including two during the first trimester, one specifically to date the pregnancy (gestational age determination). While the birth certificate does not include information regarding how the gestational age was determined, the fact that almost all women are covered by National Health Insurance and the high uptake of antenatal ultrasound, means that most women have confirmation of LMP estimates of gestational age.

We have used internal standardization for the Israeli birth population in an approach similar to that previously applied for the Canadian birth population and described in details by Wen *et al*. (2003)^37^. Standardized z scores for birthweight for gestational age and sex, were calculated as follows: z = (observed birthweight -mean birthweight)/standard deviations, where the mean and the standard deviations are based on sex-specific births at the observed gestational age (in completed weeks), based on our study population. To calculate corrected birthweight and to create population standards we excluded extreme values: gestational-age-specific z scores outside the range −5 and +5 for term births (gestational age equal or above 37 weeks), and outside the range −4 and +3 for preterm babies were excluded^52^ resulting in exclusion of 1,103 births and leaving 2,039,415 singleton live births (Fig. 1). After exclusion, percentiles of birthweight for gestational age and sex were calculated. There were no time trends in the percent of missing variables for singleton births that determined inclusion in the study.

### Outcome and covariates

Given the lack of consensus regarding the definition of macrosomia^2^, we used the definition of birthweight above 4000 gr and 4500 gr. The term LBW (birthweight < 2500 g and gestational age equal or above 37 weeks) does not take into account gestational age and includes preterm babies (<37 completed weeks of gestation) and infants with intrauterine growth restriction. To overcome the different causes and risks of these conditions we only studied term LBW^3^. Based on birthweight standards for gestational age and sex, we coded birthweight <10^th^ percentiles as small for gestational age (SGA) and >90^th^ percentiles as large for gestational age (LGA).

We focused our analysis on the following main outcome measures: (1) mean birthweight and (2) birthweight z scores by sex and gestational age in term births (gestational age equal or above 37 weeks); (3) term LBW (N = 52,772); (4) SGA (N = 198,876); (5) LGA (N = 199,217); (6) macrosomia, defined as birthweight >4,000 g (N = 109,233) and (7) macrosomia, defined as birthweight >4,500 g (N = 9,314).

Year of delivery was the primary exposure. We obtained information on infant and maternal variables. We categorized characteristics as follows: infant’s sex (male/female), infant’s religion as declared by the parents (Jewish/Muslim/Druze/ Christian), parity (nulliparous (parity 0), primiparous (parity 1), multiparous (parity 2–4), grand multiparous (parity 5–8), great grand (parity > 8) multiparous mothers), maternal age (15–20, 21–29, 30–35, 36–40, 41–55 years), maternal marital status (married/unmarried), maternal origin (Israel/former USSR/ America and Europe/Africa and Asia), and season of conception that was calculated based on birth date and gestational age and categorized according to Alpert categorization (spring (31/3–30/5), summer (31/5–22/9), autumn (23/9–6/12), winter (7/12–30/3))^53^. Maternal education was classified according to the country-specific coding scheme provided by ISCED-2011 into three categories: high (ISCED 4–8: postsecondary non-tertiary to doctoral or equivalent level), medium (ISCED 3: upper secondary education) and low (ISCED 01–2: preprimary to lower secondary education), using individual measures of education^54^. We could not consider maternal Body Mass Index (BMI), weight gain, pathologies (such as preeclampsia and gestational diabetes), illness, alcohol, tobacco and drug use during pregnancy, because these data are not recorded on the Israeli birth certificate.

### Statistical analysis

We present the distribution of the outcomes and the sociodemographic characteristics by year. We evaluated the changes compared to the baseline of 2000. We used multivariable linear regression models to estimate change in term birthweight (grams) and birthweight z scores. To estimate change in the risk of term LBW, macrosomia, SGA and LGA, separately for each outcome, we used multivariable logistic regression models. Estimates for LGA and SGA were compared to appropriate for gestational age (AGA, births with weight between 10^th^ to 90^th^ percentiles for sex and gestational age). We present the results from the linear regression models as the change in mean birthweight and mean birthweight z scores, and the results from the logistic regression models as odds ratios (ORs) along with their 95% confidence intervals (CIs). We evaluated the trends by using a cubic polynomial model. We modelled the trend of the outcomes (Yt) by polynomial regression, and the trend Tr(Yt) was calculated in the following polynomial form: 1) for ORs, log Tr(Yt) = a0 + a1 * t^1^ + a2 * t^2^ + a3 * t^3^, and 2) for mean birthweight and z-birthweight, Tr(Yt) = a0 + a1 * t^1^ + a2 * t^2 + ^a3 * t^3^ where t is a time variable, a0 is the intercept, a1, a2 and a3 are the coefficients. In our data, a year t = 0… 14. We present the a1, a2 and a3 as the linear, quadratic and cubic trends, respectively.

We adjusted the models for the sociodemographic covariates that were available for analysis and could potentially explain the association between the time trend and fetal growth outcomes. For all the outcomes we adjusted the models for the following covariates: infant’s sex, infant’s religion, gestational age, parity, maternal age, maternal marital status, maternal origin, season of conception and maternal education.

Missing data ranged from 0.02% (maternal age data) to 32.2% (maternal education). Maternal education is not required on the birth certificate reported to the Ministry of Health and data was available only for 1,386,566 births (67.7%). When we evaluated the pattern of missing values, 96.3% of the values were complete and only 64.3% of the births had complete data. We therefore used two approaches to deal with missing data: (1) We used a multiple imputation approach to impute the missing values in a similar approach described in detail elsewhere^55^. Briefly, we first tested whether the likelihood of missing data was associated with the outcomes (t-test and chi-test) (data not shown). We used imputation models that were more general than the analyses models and imputed 35 data sets. (2) We created a category of “unknown” for factors with missing values.

We then compared the results of the complete case models, the imputed models and the models including cases with missing values as “unknown”. The complete case models were not adjusted for maternal education (95.5% of the births had complete data). Generally, the direction of the trend did not change significantly between the approaches. Thus, the results presented here include the imputed models values.

Stratified analysis to identify the possible modifying effects of maternal education and infant’s religion on the changes observed over time were conducted for the imputed dataset. Due to small numbers, the stratified analysis for maternal education was based on two categories (high (ISCED 4–8) and medium and low (ISCED 01–3)) and two categories for infant’s religion (Jewish and Muslim).

The statistical analyses were conducted with SPSS 23 (SPSS Inc.,Chicago, IL).

### Ethical approval

All methods were performed in accordance with the relevant guidelines and regulations. The study was approved by the Ethics Committee of the Israeli Ministry of Health. Approvals from the Public Health Services as well as from the legal advisor in the Israeli Ministry of Health and the Ministry of Interior were also obtained.

### Data availability

The data that support the findings of this study are available from the Israeli Ministry of Health and the Israeli Ministry of Interior but restrictions apply to the availability of these data, which were used under license for the current study, and so are not publicly available. Data are however available from the authors upon reasonable request and with permission of the Israeli Ministry of Health and the Ministry of Interior.

## Electronic supplementary material

Supplementary Information 
