# Supplementary material for: Trends in Fetal Growth Between 2000 to 2014 in Singleton Live Births from Israel
Source: Sci Rep. 2018 Jan 18;8:1089. doi: 10.1038/s41598-018-19396-w (PMC5773590; doi:10.1038/s41598-018-19396-w)
Supplement: Supplementary file 1 — Supplementary Information [file 41598_2018_19396_MOESM1_ESM.pdf]

SUPPLEMENTARY MATERIAL

Manuscript title:" Trends in Fetal Growth Between 2000 to 2014 in Singleton Live Births from Israel"

Authors' full names: Keren Agay-Shay, Mary Rudolf, Lisa Rubin Ziona Haklai, Itamar Grotto

TABLE OF CONTENTS

Table 1S: Trends in birthweight and abnormal fetal weight, registry based studies since the beginning of the millennium…………………………..…...……..2

Table 2S: Gestational age distribution, by year……………………………………………………………………………………………….7

Table 3S: Crude changes in mean birthweight and z-birthweight for term births and ORs (Odds ratio) and 95% Confidence intervals (95% CI) for term LBW, Macrosomia births and SGA and LGA compared to AGA by year of birth compared to the baseline of year 2000, 2000-2014, Israel… ………………………8

Table 4S: Adjusted changes in mean birthweight and z-birthweight for term births and ORs (Odds ratio) and 95% Confidence intervals (95% CI) for term LBW, Macrosomia births and SGA and LGA compared to AGA by year of birth compared to the baseline of year 2000, imputed data, 2000-2014, Israel………..9

Table 5S: Adjusted changes in mean birthweight and z-birthweight for term births and ORs (Odds ratio) and 95% Confidence intervals (95% CI) for term LBW, Macrosomia births and SGA and LGA compared to AGA by year of birth compared to the baseline of year 2000, complete case models, 2000-2014, Israel.…….......10

Table 6S: Adjusted changes in mean birthweight and z-birthweight for term births and ORs(Odds ratio) and 95% Confidence intervals(95% CI) for term LBW, Macrosomia births and SGA and LGA compared to AGA by year of birth compared to the baseline of year 2000, 2000-2014, Israel, "unknown" category …….….…….11

Figure 1S: Changes in proportion (%) of maternal and child characteristics in singleton births, by year (N=2,039,415)……...………..…………………...….12

Figure 2S: Adjusted change in mean birth weight for term births and 95% confidence intervals (95% CI) compared to the baseline of year 2000, by year of birth, by religion and by educational category, imputed data ….…………………………………………………………………………..……...….13

Table 1S: Trends in birthweight and abnormal fetal weight, registry based studies since the beginning of the millennium.

Author, Year	Years of the study	Country	Region, hospital based/ registry based and Population size	Outcomes	Adjustment	Trend
Martin JA 2000-2014 [22-36]	2000-2014	USA	Approximately 4 million births annually and approximately total of 60 million births. Registry based national vital statistics, all births.	LBW<br>VLBW<br>Macrosomia (4000 gr)	Crude	The LBW rate increased from 2000 until 2006 (7.57-8.26%) and then decreased until 2012 (7.99%), and slightly increased until 2014 (8.0%).<br>The VLBW rate increased from 2000 (1.43%) until 2005(1.49%) and was stable until 2007 and since then decreased until 2014 (1.40%).<br>The Macrosomia rate decreased from 2000 (9.9%) until 2010(7.6%) and increase until 2014(8.0%).<br>Generally, rates of LBW increased, rates of VLBW and Macrosomia decreased.
					Stratification by ethnicity.	The LBW rates and VLBW for non-Hispanic white increased from 2000(6.6%, 1.14%) until a peak during 2006 for LBW (7.32%) and 2005 for VLBW(1.21%) and declined until 2014(LBW - 6.96%, VLBW-1.1%,).<br>Similar pattern for non-Hispanic Black. Increase trend from 2000 rate(LBW-13.13%,VLBW-3.1%) to a peak at 2005 (LBW-14.02%,VLBW-3.27%) and decline until 2013.<br>For Hispanic, rates increased from 2000 ((LBW-13.13%,VLBW-3.1%)until 2014 (LBW-13.13%,VLBW-3.1%) with small fluctuation.<br>For Macrosomia, rates for non-Hispanic White decreased from 2000(11.7%) until 2007(9.1%) and was stable until 2010 and slightly increased until 2014(9.7%). Similar pattern was for non-Hispanic Black from 2000 (5.3%) with lowest peak during 2008(4.1%) and increase trend from 2010 until 2014(4.5%). For Hispanic a decrease trend was observed from 2000(9.0%) with lowest peak during 2010(7.0%) and increase until 2014(7.2%).<br>Generally, for all Races and Origins, Macrosomia rates during 2014 were lower compared to rates during 2000.
de Souza Buriol, 2016 [38]	1999-2011	Brazil	11,200,255 births from 26 state capitals and Brasilia (the federal capital)	LBW	- Crude	The LBW rate was 8 %, and it was stable during the period.
					- Adjusted to **gestational age,** maternal age, maternal educational level, number of antenatal visits, type of delivery	- In the full model, the risk of LBW decreased in all regions each year.

Takemoto, 2016 [12]	1979-2010	Japan	5,106,085 births during 1979, 1990, 2000, 2010	Mean BW	Stratification by infant sex	The mean BW for male infants decreased from 3,193 ± 442 g in 1979 to 3,059 ± 427 g in 2010, and the mean BW for female infants decreased from 3,109 ± 426 g in 1979 to 2,974 ± 409 g in 2010.
				LBW	Stratification by maternal age	The prevalence of LBW infants increased from 4.5% in 1979 to 8.3% in 2010, and the prevalence of term LBW infants increased from 2.7% in 1979 to 5.3% in 2010
				LBW	Adjusted odds ratio of women ≥35 years compared to women <35 years. Adjusted to infant sex, birthplace (urban area, rural area).	AORs for LBW infants significantly decreased from 2.06 (95% CI: 1.95–2.18) to 1.34 (95% CI: 1.31–1.37) among nulliparous women and from 1.90 (95% CI: 1.83–1.97) to 1.21 (95% CI: 1.18–1.24) among multiparous women.
Morisaki, 2013 [13]	2000-2008	USA, Utah and Southeast Idaho.	219,694 singletons term births. From medical records of 21 hospitals affiliated with Intermountain Healthcare	Mean BW<br>LGA<br>SGA.	Crude	Over the period mean BW(3410g to 3383g), and LGA (9.0% to 7.4%) both decreased, whereas<br>SGA increased (7.5% to 8.2%).
					Adjusted to: **gestational age**, maternal age, family status, prenatal care, race/ethnicity, parity, smoking, diabetes, eclampsia, BMI, wait gain, delivery type	Similar to crude:<br>BW for gestational age decreased by 36g (95% CI: −31, −42). The estimated ORs for SGA was 1.12 (95% CI: 1.06, 1.19) and for LGA 0.77 (95% CI: 0.73, 0.82), for delivery in 2008 compared to delivery in 2000.
Lopez & Bréart, 2012 [37]	1991-2008	Chile	4,559,917 All live births (singleton and multiple) from national registry	BW<br>LBW<br>Macrosomia (4000gr)	Crude	LBW births increased in 13.8% (from 4.62% in 1991 to 5.27% in 2008). Macrosomia rates remained stable.
					Stratified by BW and gestational age categories	An increase trends in gestational age and BW in preterm birth and LBW births, a reduction in term and postterm births and a stable level of BW above 4000 grams.
Lu, 2011 [17]	1994-2005	Southeastern China	593,728 term and posttem births, perinatal health care surveillance system	Mean BW<br>SGA<br>LGA<br>Macrosomia	Crude	Mean BW for all term and postterm infants increased from 3296 g in 1994 to 3378 g in 2000, then decreased to 3369 g in 2005.<br>The incidence of LGA increased significantly from 13.72% in 1994 to 18.98% in 2005.<br>The percentage of SGA infants declined steadily from 11.95% in 1994 to 7.00% in 2005.
				Mean BW	Stratification by gestational age	Mean BW increased. Increments of BW differed considerably by gestational age. Mean BW rose the most at 38-41 weeks (more than 80 g) from 1994 to 2005.

				LGA	Adjustment for gestational age, gender and parity	Adjustment enlarged the yearly effect between 1994 and 2005.
Ferré, 2011 [14]	1991-2004	US	8,162,756 Singleton non-Hispanic black from national Statistics Vital Records	Mean BW<br>LBW	Adjustment for maternal age, education, birth place, metropolitan county residence, marital status, and any tobacco smoking during pregnancy	Mean BW increased from 3,121g in 1991 to 3,136g in 2001 and then decreased to 3,115 g in 2004.<br>The LBW rate declined from 1991 (12.15%) to 1996 (11.55%). Between 1996 and 2001, the rate declined more slowly, from 11.55% in 1996 to 11.19% in 2001. The rate then slightly increased from 11.19% in 2001 to 11.70% in 2004.
					Adjustment to first-trimester prenatal care, maternal education, and foreign birth place.	For the 1991–2001 MLBW rate decrease, adjustment reduced the observed trend.<br>Further adjustment to smoking and weight gain eliminated trend.
Diouf 2011 [39]	1972-2003	French	49,281 Singleton term births from the French national perinatal surveys of 1972, 1981, 1995, 1998 and 2003.	Mean BW<br>LGA<br>SGA	Crude	Infant BW did not change over time.<br>SGA rates decreased between 1972 and 1995 (10.6–7.5%), increased thereafter reaching 8.7% in 2003.<br>The percentage of LGA births remained around 11% until 1998, then decreased slightly to 9.9% in 2003.
					Adjusted to **gestational age**, maternal age, parity, country of origin, newborn gender and maternal smoking during pregnancy,	After adjusting the mean BW in term births increased between 1972 and 1995 and decreased between 1998 to 2003.<br>Changes in the risk of LGA and of SGA births were consistent with trend in mean BW. There was a strong decrease in SGA between 1972 and 1995, whereas between 1995 and 2003, an increase in SGA and a decrease in LGA were observed.
Zhang 2010 [16]	1992-2003	US	23,549,360 term live births singleton non-Hispanic white births	Mean BW<br>Macrosomia (4500 gr)<br>SGA<br>LGA	Crude	BW decreased by 37 g.<br>Rate of macrosomia declined.<br>SGA rates declined from 9.9% to 8.3% during 2000 and were stable until 2003(8.3%)<br>LGA rate remained relatively stable.

Donahue 2010 [15]	1990–2005	US	36,827,828 term singletons born to U.S. resident mothers aged 18 years and older	Mean BW<br>SGA<br>LGA	Crude<br>.	Mean BW among all term singleton neonates decreased by 52g.<br>Rates of LGA remained fairly stable at about 10.3% until 2000 and thereafter steadily decreased to 8.9% in 2005.<br>The percentage of SGA remained steady from 1990-1996 at about 10.3%, declined slightly until 1999, and thereafter increased back to 10.2% in 2005.
					Adjusted to maternal age, race/ethnicity, tobacco use, marital status, timing of prenatal care initiation, maternal gestational weight gain, medical risk factors.	Decrease in mean BW by gestational age.<br>Results were generally greater after adjustment
Hadfield 2009 [20]	1990–2005	New South Wales	1,273,924 live-born singletons term births	BW<br>LGA<br>Macrosomia (> 4000 g),<br>Macrosomia (> 4500 g),	Stratification and adjustment to Infant sex, maternal age, parity, smoking during pregnancy, pre-existing diabetes, gestational diabetes, hypertensive disorders during pregnancy, and maternal region of birth and year of birth.	Mean BW increased significantly by 23 g for boys and 25 g for girls.<br>For male infants: the rates of macrosomia (4000g) increased from 14.3% to 15.8% (10.5% increase), and Macrosomia rate (4500 g) increased from 2.2% to 2.4% (9.0% increase); rates of LGA increased from 9.2% to 10.8% (17.9% increase).<br>For female infants: the rate of Macrosomia (4000g) increased from 8.3% to 9.5% (15.2% increase), and the rates for Macrosomia (4500g) increased from 1.0% to 1.2% (20.0% increase); rates for LGA increased from 9.1% to 11.0% (21.0% increase).
Lahmann, 2009 [19]	from 1988 to 2005	Queensland, Australia,	831,375 singleton livebirths from national registry	Mean BW<br>Macrosomia (BW>4000g, 5000g)	Crude	Mean BW increased during the 17-year interval by ~1.9 g/year corresponding to a total increase of approximately 32 g. The small increase in BW was relatively steady until 2001 where after a slight decrease in BW was observed.<br>The proportion of macrosomia (>4000 g) significantly increased. (but after 2000 rates decreased)<br>Proportion of Macrosomia(>5000g) not significantly increased.
					Adjustment and stratification to **gestational age**, sex,	The increase in BW was 4.5 g per year.<br>Among Indigenous infants BW did not change significantly for boys and for girls.

					Indigenous status, and maternal age.	The proportion of Macrosomia (>4000 g) increased. In term infants, macrosomia significantly increased in both non- Indigenous boys and girls, but not in Indigenous boys and girls.<br>On average, the proportion of infants with extremely high BW > 5000 g did not significantly increase.
Schiessl, 2009 [18]	2000–2007	Bavaria, Germany	695,707 singleton term births from registry	Mean BW Macrosomia (4000g)	Crude	Slight decreases in mean BW and in the percentage of Macrosomia was observed.
					Stratification by primiparous	A stratified showed similar trends.
Schack-Nielsen, 2006 [21]	1973-2003	Denmark	All single live births 1,863,456	Mean BW Macrosomia (4000,4500, 5000,5500g)	Crude	Mean BW increased steadily during the period (160 grams; equivalent to 5 g/yr).<br>Increase in percentage of Macrosomia.
					Adjusted to decreasing gestational age, maternal age and maternal smoking prevalence (only data after 1991)	There was still an increase in BW of 4 g/yr.
					Stratification by gestational age, sex and smoking status (after 1991.	Among term infants, the increase in BW per year remained almost unchanged.<br>The secular increase in BW was larger among girls than among boys.<br>Stratifying for smoking showed that the secular increase in BW was slightly higher among offspring of smoking compared with non-smoking mothers.

AGA-appropriate gestational age(10th to 90th percentile); BW-birthweight; BMI- body mass index, CI- confidence interval; LBW- Low birthweight(birthweight under 2500 gr); LGA-large for gestational age by sex(above 90th percentile); MLBW –medium LBW (1500 gr <= BW <=2500 gr); NBW- BW above 2500 gr; ORs-odds ratio; SGA-small for gestational age by sex (under 10th percentile); Term births- gestational age equal or above 37 weeks; VLBW- very low birthweight (under 1500 gr)

Table 2S: Gestational age distribution, by year, 2000-2014, Israel(N=2,039,415).

Gestational age	2000	2001	2002	2003	2004	2005	2006	2007	2008	2009	2010	2011	2012	2013	2014
**22**	0.01	0.01	0.01	0.01	0.00	0.00	0.00	0.01	0.01	0.01	0.01	0.01	0.01	0.00	0.00
**23**	0.01	0.02	0.02	0.02	0.01	0.02	0.01	0.02	0.01	0.02	0.03	0.02	0.02	0.02	0.01
**24**	0.03	0.03	0.02	0.03	0.03	0.02	0.02	0.02	0.03	0.03	0.03	0.02	0.02	0.02	0.02
**25**	0.04	0.04	0.04	0.05	0.04	0.04	0.02	0.04	0.04	0.04	0.03	0.03	0.02	0.04	0.03
**26**	0.06	0.05	0.06	0.04	0.05	0.04	0.04	0.04	0.05	0.04	0.04	0.05	0.04	0.04	0.03
**27**	0.07	0.06	0.06	0.06	0.07	0.06	0.06	0.04	0.05	0.05	0.05	0.05	0.05	0.05	0.05
**28**	0.09	0.09	0.08	0.07	0.07	0.08	0.07	0.06	0.06	0.06	0.07	0.06	0.07	0.05	0.06
**29**	0.11	0.08	0.09	0.08	0.07	0.10	0.08	0.08	0.08	0.08	0.08	0.08	0.07	0.07	0.07
**30**	0.13	0.13	0.12	0.13	0.13	0.11	0.10	0.10	0.11	0.10	0.10	0.10	0.11	0.10	0.09
**31**	0.14	0.12	0.14	0.14	0.16	0.14	0.16	0.14	0.13	0.13	0.13	0.13	0.14	0.12	0.14
**32**	0.23	0.24	0.22	0.29	0.38	0.26	0.26	0.24	0.22	0.20	0.24	0.23	0.21	0.21	0.22
**33**	0.32	0.35	0.34	0.34	0.34	0.33	0.31	0.33	0.30	0.30	0.31	0.32	0.29	0.29	0.30
**34**	0.61	0.58	0.65	0.67	0.68	0.70	0.66	0.66	0.65	0.62	0.62	0.64	0.60	0.61	0.63
**35**	1.12	1.15	1.19	1.15	1.22	1.19	1.18	1.17	1.16	1.19	1.21	1.07	1.09	1.06	1.04
**36**	2.38	2.39	2.53	2.56	2.82	2.59	2.57	2.75	2.57	2.51	2.47	2.38	2.32	2.35	2.31
**37**	5.35	5.68	5.72	5.84	6.04	6.58	6.46	6.78	6.60	6.69	6.69	6.48	6.45	6.64	6.52
**38**	12.53	13.41	13.65	13.89	14.44	15.32	15.76	15.85	16.14	16.40	16.15	16.31	16.21	16.53	17.06
**39**	21.88	22.86	23.15	23.22	23.23	24.42	24.37	24.86	24.80	24.93	24.62	25.41	25.40	26.01	26.27
**40**	32.56	31.86	31.52	32.08	31.59	30.48	31.31	30.49	30.15	30.02	29.24	28.29	28.45	28.56	28.36
**41**	16.78	16.37	16.08	15.39	14.91	14.55	13.84	13.79	14.19	13.95	14.73	15.17	15.26	14.57	14.25
**42**	5.55	4.48	4.31	3.92	3.72	2.98	2.72	2.54	2.64	2.61	3.16	3.14	3.17	2.67	2.52

Percentages sum up to 100% in each column, except in cases of rounding.

Table 3S: Crude changes in mean birthweight and z-birthweight for term births and ORs (Odds ratio) and 95% Confidence intervals (95% CI) for term LBW, Macrosomia births and SGA and LGA compared to AGA by year of birth compared to the baseline of year 2000, imputed data, 2000-2014, Israel.

Year of birth	Term birthweight			Term Z birthweight			Term LBW			Macrosomia 4000 gr			Macrosomia 4500 gr			LGA compared to AGA			SGA compared to AGA		
	Change in mean birth weight	95% CI		Change in mean z-birth weight	95% CI		ORs	95% CI		ORs	95% CI		ORs	95% CI		ORs	95% CI		ORs	95% CI	
		Lower	Upper		Lower	Upper		Lower	Upper		Lower	Upper		Lower	Upper		Lower	Upper		Lower	Upper
**2000**	Reference			Reference			Reference			Reference			Reference			Reference			Reference		
**2001**	-1.01	-4.63	2.61	0.02	0.01	0.02	0.99	0.94	1.04	0.99	0.96	1.02	0.96	0.86	1.07	1.00	0.97	1.02	0.95	0.93	0.98
**2002**	-2.99	-6.59	0.60	0.02	0.01	0.03	0.94	0.89	0.99	0.96	0.93	0.99	0.88	0.79	0.98	0.98	0.96	1.01	0.92	0.90	0.94
**2003**	-9.09	-12.67	-5.52	0.01	0.00	0.02	0.97	0.92	1.02	0.92	0.88	0.95	0.90	0.80	1.00	0.96	0.94	0.99	0.93	0.91	0.96
**2004**	-9.86	-13.43	-6.28	0.02	0.01	0.02	0.98	0.93	1.03	0.90	0.87	0.93	0.87	0.78	0.97	0.98	0.96	1.01	0.91	0.88	0.93
**2005**	-11.87	-15.44	-8.30	0.03	0.02	0.04	1.01	0.96	1.06	0.89	0.86	0.92	0.78	0.70	0.87	0.97	0.94	1.00	0.88	0.85	0.90
**2006**	-15.80	-19.35	-12.26	0.02	0.02	0.03	0.98	0.94	1.03	0.88	0.85	0.91	0.76	0.68	0.85	0.97	0.95	1.00	0.90	0.87	0.92
**2007**	-21.59	-25.11	-18.07	0.02	0.01	0.03	0.99	0.94	1.04	0.84	0.81	0.87	0.71	0.63	0.79	0.94	0.91	0.96	0.90	0.87	0.92
**2008**	-23.16	-26.64	-19.68	0.01	0.00	0.02	0.97	0.92	1.02	0.82	0.79	0.85	0.70	0.63	0.78	0.90	0.88	0.92	0.89	0.87	0.91
**2009**	-22.07	-25.52	-18.62	0.02	0.01	0.03	0.95	0.90	0.99	0.85	0.82	0.87	0.71	0.64	0.80	0.92	0.90	0.94	0.88	0.86	0.90
**2010**	-18.25	-21.69	-14.82	0.02	0.01	0.03	0.93	0.89	0.98	0.86	0.83	0.89	0.69	0.62	0.77	0.91	0.89	0.94	0.87	0.85	0.90
**2011**	-22.55	-25.96	-19.13	0.01	0.00	0.02	0.95	0.91	1.00	0.84	0.81	0.87	0.72	0.64	0.80	0.90	0.88	0.92	0.88	0.86	0.91
**2012**	-19.22	-22.62	-15.82	0.02	0.01	0.02	0.96	0.92	1.01	0.85	0.83	0.88	0.70	0.63	0.78	0.90	0.88	0.93	0.88	0.86	0.91
**2013**	-18.12	-21.51	-14.73	0.03	0.02	0.04	0.92	0.88	0.97	0.84	0.81	0.87	0.72	0.65	0.80	0.90	0.88	0.92	0.84	0.82	0.86
**2014**	-16.25	-19.62	-12.88	0.04	0.03	0.04	0.91	0.87	0.95	0.84	0.82	0.87	0.68	0.61	0.76	0.91	0.89	0.93	0.83	0.81	0.85
**Linear trend ***	0.011	0.008	0.014	-2.881	-4.179	-1.583	0.997	0.979	1.015	0.963	0.951	0.975	0.945	0.908	0.985	1.002	0.992	1.012	0.958	0.949	0.967
**Quadratic trend ***	-0.002	-0.002	-0.001	-0.111	-0.327	0.104	1.001	0.998	1.004	1.002	1.000	1.004	1.001	0.994	1.008	0.998	0.996	0.999	1.006	1.004	1.007
**Cubic trend***	0.000	0.000	0.000	0.017	0.007	0.027	1.000	1.000	1.000	1.000	1.000	1.000	1.000	1.000	1.000	1.000	1.000	1.000	1.000	1.000	1.000

AGA-appropriate gestational age (10th to 90th percentile); LBW- Low birthweight (birthweight<2500 gr), LGA-large for gestational age by sex(above 90th percentile), Macrosomia(birthweight> 4000 gr and birthweight>4500 gram), SGA- small for gestational age by sex (under 10th percentile);Term births-births at gestational age equal or above 37 weeks *Polynomial models were adjusted to the three trend component in a single model.

Table 4S: Adjusted* changes in mean birthweight and z-birthweight for term births and ORs (Odds ratio) and 95% Confidence intervals (95% CI) for term LBW, Macrosomia births and SGA and LGA compared to AGA by year of birth compared to the baseline of year 2000, imputed data, 2000-2014, Israel.

Year of birth	Term birthweight			Term Z birthweight			Term LBW			Macrosomia 4000 gr			Macrosomia 4500 gr			LGA compared to AGA			SGA compared to AGA		
	Change in mean birth weight	95% CI		Change in mean z-birth weight	95% CI		ORs	95% CI		ORs	95% CI		ORs	95% CI		ORs	95% CI		ORs	95% CI	
		Lower	Upper		Lower	Upper		Lower	Upper		Lower	Upper		Lower	Upper		Lower	Upper		Lower	Upper
**2000**	Reference			Reference			Reference			Reference			Reference			Reference			Reference		
**2001**	5.45	2.17	8.74	0.01	0.00	0.02	0.95	0.90	1.00	1.01	0.98	1.05	0.98	0.92	1.03	0.99	0.96	1.02	0.96	0.94	0.99
**2002**	4.72	1.46	7.98	0.01	0.00	0.02	0.90	0.85	0.95	0.99	0.96	1.03	0.91	0.82	1.01	0.97	0.95	1.00	0.93	0.91	0.96
**2003**	1.43	-1.82	4.68	0.00	-0.01	0.01	0.92	0.87	0.96	0.96	0.93	0.99	0.94	0.84	1.05	0.95	0.93	0.98	0.95	0.93	0.98
**2004**	3.65	0.40	6.90	0.01	0.00	0.01	0.91	0.87	0.96	0.96	0.92	0.99	0.93	0.84	1.04	0.97	0.95	1.00	0.93	0.90	0.95
**2005**	8.06	4.81	11.30	0.02	0.01	0.02	0.90	0.85	0.95	0.98	0.94	1.01	0.86	0.77	0.96	0.96	0.93	0.98	0.91	0.88	0.93
**2006**	5.38	2.16	8.60	0.01	0.00	0.02	0.89	0.84	0.93	0.97	0.94	1.01	0.85	0.76	0.95	0.96	0.93	0.98	0.94	0.91	0.96
**2007**	1.43	-1.77	4.64	0.00	-0.01	0.01	0.88	0.83	0.92	0.94	0.90	0.97	0.80	0.72	0.90	0.92	0.89	0.94	0.94	0.92	0.96
**2008**	-1.02	-4.19	2.15	-0.01	-0.02	0.00	0.86	0.82	0.91	0.90	0.87	0.94	0.79	0.70	0.88	0.88	0.86	0.90	0.93	0.91	0.96
**2009**	0.77	-2.37	3.92	0.00	-0.01	0.00	0.84	0.80	0.89	0.94	0.91	0.97	0.81	0.72	0.90	0.90	0.88	0.92	0.93	0.90	0.95
**2010**	-0.51	-3.65	2.63	0.00	-0.01	0.00	0.84	0.80	0.89	0.93	0.90	0.96	0.76	0.68	0.85	0.89	0.87	0.91	0.93	0.90	0.95
**2011**	-4.94	-8.06	-1.82	-0.02	-0.02	-0.01	0.87	0.82	0.91	0.90	0.87	0.94	0.79	0.71	0.88	0.87	0.85	0.90	0.94	0.92	0.96
**2012**	-2.95	-6.06	0.16	-0.01	-0.02	0.00	0.88	0.84	0.93	0.91	0.88	0.94	0.76	0.68	0.85	0.87	0.85	0.90	0.94	0.92	0.97
**2013**	2.06	-1.04	5.17	0.00	-0.01	0.01	0.83	0.79	0.87	0.92	0.89	0.95	0.80	0.72	0.89	0.87	0.85	0.89	0.89	0.87	0.92
**2014**	5.96	2.88	9.05	0.01	0.00	0.02	0.81	0.77	0.85	0.93	0.90	0.96	0.76	0.68	0.85	0.88	0.86	0.90	0.89	0.86	0.91
**Linear trend ****	4.192	3.015	5.369	0.008	0.005	0.011	0.9613	0.9434	0.9795	0.9953	0.9826	1.0082	0.9764	0.9374	1.0170	0.9995	0.9898	1.0094	0.9639	0.9545	0.9733
**Quadratic trend ****	-0.852	-1.047	-0.656	-0.002	-0.002	-0.001	1.0043	1.0012	1.0075	0.9987	0.9966	1.0008	0.9985	0.9916	1.0054	0.9976	0.9961	0.9992	1.0057	1.0041	1.0073
**Cubic trend****	0.041	0.032	0.050	0.000	0.000	0.000	0.9998	0.9997	1.0000	1.0001	1.0000	1.0002	1.0001	0.9998	1.0005	1.0001	1.0001	1.0002	0.9997	0.9997	0.9998

AGA-appropriate gestational age (10th to 90th percentile); LBW- Low birthweight(birthweight<2500 gr), LGA-large for gestational age by sex(above 90th percentile), Macrosomia(birthweight> 4000 gr and birthweight>4500 gram), SGA- small for gestational age by sex (under 10th percentile);Term births-births at gestational age equal or above 37 weeks *All models were adjusted to gestational age, child sex, child religion, season of conception, maternal parity, maternal origin of birth, maternal family status and maternal age, maternal education; **Polynomial models were adjusted to the three trend component in a single model.

Table 5S: Adjusted* changes in mean birthweight and z-birthweight for term births and ORs (Odds ratio) and 95% Confidence intervals (95% CI) for term LBW, Macrosomia births and SGA and LGA compared to AGA by year of birth compared to the baseline of year 2000, complete case models, 2000-2014, Israel.

Year of birth	Term birthweight			Term Z birthweight			Term LBW			Macrosomia 4000 gr			Macrosomia 4500 gr			LGA compared to AGA			SGA compared to AGA		
	Change in mean birth weight	95% CI		Change in mean z-birth weight	95% CI		ORs	95% CI		ORs	95% CI		ORs	95% CI		ORs	95% CI		ORs	95% CI	
		Lower	Upper		Lower	Upper		Lower	Upper		Lower	Upper		Lower	Upper		Lower	Upper		Lower	Upper
**2000**	Reference			Reference			Reference			Reference			Reference			Reference			Reference		
**2001**	6.64	3.29	10.00	0.01	0.01	0.02	0.95	0.90	1.00	1.02	0.98	1.05	0.96	0.86	1.08	0.99	0.96	1.01	0.96	0.93	0.98
**2002**	6.44	3.11	9.77	0.01	0.01	0.02	0.89	0.84	0.94	0.99	0.96	1.03	0.89	0.80	1.00	0.97	0.95	1.00	0.92	0.90	0.95
**2003**	3.80	0.49	7.11	0.01	0.00	0.01	0.90	0.85	0.95	0.96	0.93	1.00	0.94	0.84	1.05	0.95	0.93	0.98	0.93	0.91	0.96
**2004**	6.95	3.63	10.26	0.01	0.01	0.02	0.89	0.84	0.93	0.96	0.93	1.00	0.91	0.81	1.02	0.98	0.95	1.00	0.90	0.88	0.93
**2005**	11.90	8.59	15.21	0.03	0.02	0.03	0.87	0.83	0.92	0.98	0.95	1.02	0.85	0.75	0.95	0.95	0.93	0.98	0.88	0.86	0.91
**2006**	9.44	6.16	12.73	0.02	0.01	0.03	0.86	0.81	0.90	0.98	0.94	1.01	0.85	0.75	0.95	0.96	0.93	0.98	0.91	0.88	0.93
**2007**	6.71	3.46	9.97	0.01	0.00	0.02	0.85	0.80	0.89	0.95	0.92	0.99	0.79	0.71	0.89	0.92	0.90	0.95	0.91	0.88	0.93
**2008**	5.23	2.01	8.46	0.01	0.00	0.02	0.83	0.79	0.87	0.92	0.89	0.95	0.79	0.70	0.89	0.89	0.86	0.91	0.89	0.87	0.92
**2009**	7.58	4.39	10.78	0.01	0.01	0.02	0.80	0.76	0.84	0.95	0.92	0.99	0.80	0.72	0.90	0.91	0.88	0.93	0.88	0.86	0.91
**2010**	6.81	3.63	10.00	0.01	0.01	0.02	0.80	0.76	0.84	0.94	0.91	0.98	0.76	0.68	0.86	0.90	0.88	0.93	0.88	0.86	0.90
**2011**	2.37	-0.80	5.54	0.00	-0.01	0.01	0.82	0.78	0.86	0.92	0.88	0.95	0.77	0.69	0.86	0.88	0.86	0.90	0.89	0.87	0.92
**2012**	4.44	1.28	7.59	0.01	0.00	0.02	0.84	0.80	0.88	0.93	0.90	0.96	0.76	0.68	0.85	0.88	0.86	0.91	0.90	0.87	0.92
**2013**	10.20	7.06	13.35	0.02	0.01	0.03	0.78	0.74	0.82	0.94	0.90	0.97	0.80	0.71	0.89	0.88	0.86	0.91	0.85	0.82	0.87
**2014**	13.62	10.50	16.75	0.03	0.02	0.04	0.77	0.73	0.81	0.95	0.91	0.98	0.76	0.67	0.84	0.89	0.87	0.91	0.84	0.82	0.86
**Linear trend ****	4.806	3.603	6.008	0.010	0.007	0.013	0.955	0.937	0.974	0.995	0.982	1.008	0.971	0.931	1.013	0.999	0.989	1.009	0.958	0.949	0.968
**Quadratic trend ****	-0.813	-1.012	-0.614	-0.002	-0.002	-0.001	1.004	1.001	1.008	0.999	0.997	1.001	1.000	0.992	1.007	0.998	0.996	1.000	1.006	1.004	1.007
**Cubic trend****	0.038	0.029	0.047	0.000	0.000	0.000	1.000	1.000	1.000	1.000	1.000	1.000	1.000	1.000	1.000	1.000	1.000	1.000	1.000	1.000	1.000

AGA-appropriate gestational age (10th to 90th percentile); LBW- Low birthweight(birthweight<2500 gr), LGA-large for gestational age by sex(above 90th percentile), Macrosomia(birthweight> 4000 gr and birthweight>4500 gram), SGA- small for gestational age by sex (under 10th percentile);Term births-births at gestational age equal or above 37 weeks *All models were adjusted to gestational age, child sex, child religion, season of conception, maternal parity, maternal origin of birth, maternal family status and maternal age; **Polynomial models were adjusted to the three trend component in a single model.

Table 6S: Adjusted* changes in mean birthweight and z-birthweight for term births and ORs(odds ratio) and 95% Confidence intervals(95% CI) for term LBW, Macrosomia births and SGA and LGA compared to AGA by year of birth compared to the baseline of year 2000, 2000-2014, Israel.

Year of birth	Term weight			Term Z birthweight			Term LBW			Macrosomia 4000 gr			Macrosomia 4500 gr			LGA compared to AGA			SGA compared to AGA		
	Change in mean birth weight	95% CI		Change in mean z-birth weight	95% CI		ORs	95% CI		ORs	95% CI		ORs	95% CI		ORs	95% CI		ORs	95% CI	
		Lower	Upper		Lower	Upper		Lower	Upper		Lower	Upper		Lower	Upper		Lower	Upper		Lower	Upper
**2000**	Reference			Reference			Reference			Reference			Reference			Reference			Reference		
**2001**	6.15	2.86	9.43	0.01	0.01	0.02	0.95	0.90	1.00	1.02	0.98	1.05	0.98	0.88	1.09	0.99	0.96	1.02	0.96	0.93	0.98
**2002**	6.27	3.01	9.54	0.01	0.01	0.02	0.89	0.85	0.94	1.00	0.96	1.03	0.91	0.82	1.02	0.98	0.95	1.00	0.93	0.90	0.95
**2003**	3.92	0.67	7.17	0.01	0.00	0.01	0.90	0.86	0.95	0.97	0.94	1.00	0.95	0.85	1.05	0.96	0.93	0.99	0.94	0.91	0.96
**2004**	7.80	4.55	11.05	0.02	0.01	0.02	0.89	0.84	0.94	0.97	0.94	1.01	0.94	0.85	1.05	0.98	0.96	1.01	0.91	0.88	0.93
**2005**	12.65	9.41	15.90	0.03	0.02	0.03	0.87	0.83	0.92	0.99	0.96	1.03	0.87	0.77	0.97	0.97	0.94	0.99	0.88	0.86	0.91
**2006**	10.71	7.49	13.93	0.02	0.01	0.03	0.86	0.81	0.90	0.99	0.96	1.03	0.86	0.77	0.96	0.97	0.94	1.00	0.91	0.88	0.93
**2007**	3.58	0.38	6.78	0.00	0.00	0.01	0.86	0.82	0.90	0.94	0.91	0.97	0.80	0.72	0.90	0.92	0.90	0.95	0.92	0.90	0.95
**2008**	0.96	-2.21	4.13	0.00	-0.01	0.01	0.85	0.81	0.89	0.91	0.88	0.94	0.79	0.70	0.88	0.88	0.86	0.91	0.92	0.90	0.94
**2009**	3.41	0.26	6.56	0.00	0.00	0.01	0.82	0.78	0.87	0.95	0.91	0.98	0.81	0.73	0.90	0.90	0.88	0.93	0.91	0.88	0.93
**2010**	3.25	0.12	6.39	0.00	0.00	0.01	0.82	0.78	0.86	0.94	0.91	0.97	0.76	0.68	0.85	0.90	0.87	0.92	0.90	0.88	0.93
**2011**	-0.18	-3.30	2.94	0.00	-0.01	0.00	0.84	0.80	0.88	0.92	0.89	0.95	0.79	0.71	0.89	0.88	0.86	0.91	0.91	0.89	0.93
**2012**	0.74	-2.36	3.85	0.00	-0.01	0.01	0.86	0.82	0.90	0.92	0.89	0.96	0.76	0.68	0.85	0.88	0.86	0.90	0.92	0.89	0.94
**2013**	6.07	2.97	9.17	0.01	0.00	0.02	0.81	0.77	0.85	0.93	0.90	0.96	0.80	0.72	0.90	0.88	0.86	0.90	0.87	0.85	0.89
**2014**	10.75	7.68	13.83	0.02	0.01	0.03	0.78	0.75	0.82	0.94	0.91	0.98	0.77	0.69	0.86	0.89	0.87	0.91	0.86	0.84	0.88
**Linear trend ****	5.909	4.729	7.088	0.012	0.010	0.015	0.951	0.933	0.969	1.002	0.990	1.015	0.981	0.942	1.022	1.005	0.995	1.015	0.956	0.946	0.965
**Quadratic trend ****	-1.084	-1.279	-0.888	-0.002	-0.003	-0.002	1.006	1.002	1.009	0.998	0.996	1.000	0.998	0.991	1.005	0.997	0.995	0.998	1.007	1.005	1.008
**Cubic trend****	0.051	0.042	0.060	0.000	0.000	0.000	1.000	1.000	1.000	1.000	1.000	1.000	1.000	1.000	1.000	1.000	1.000	1.000	1.000	1.000	1.000

AGA-appropriate gestational age (10th to 90th percentile); LBW- Low birthweight(birthweight<2500 gr), LGA-large for gestational age by sex(above 90th percentile), Macrosomia(birthweight> 4000 gr and birthweight>4500 gram), SGA- small for gestational age by sex (under 10th percentile);Term births-births at gestational age equal or above 37 weeks *All models were adjusted to gestational age, child sex, child religion, season of conception, maternal parity, maternal origin of birth, maternal family status maternal age and maternal education; models included "unknown" category **Polynomial models were adjusted to the three trend component in a single model.

Figure 1S: Changes in proportion (%) of maternal and child characteristics in singleton births, by year (N=2,039,415), 2000-2014, Israel.

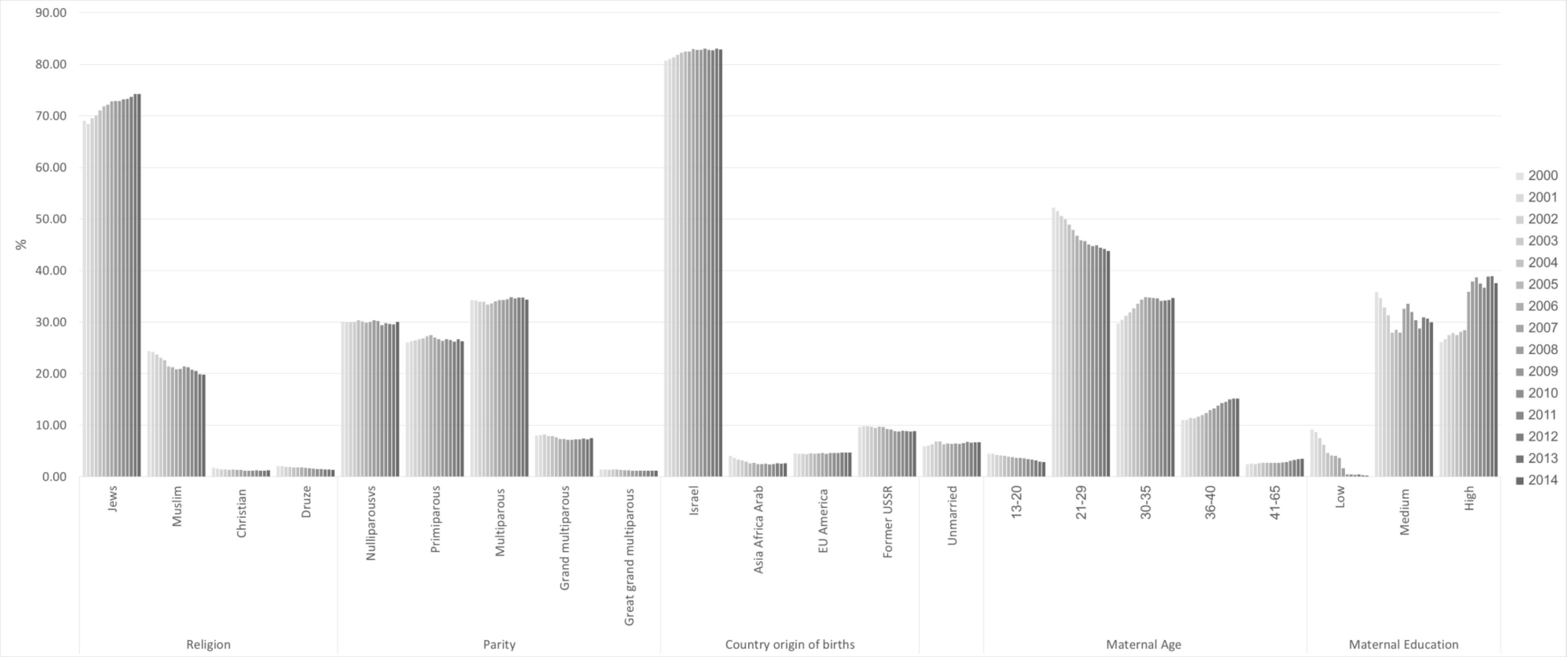

Figure 2S: Adjusted* changes in mean birthweight for term births and 95% confidence intervals (95% CI) compared to the baseline of year 2000, by year of birth, by religion and by educational category.

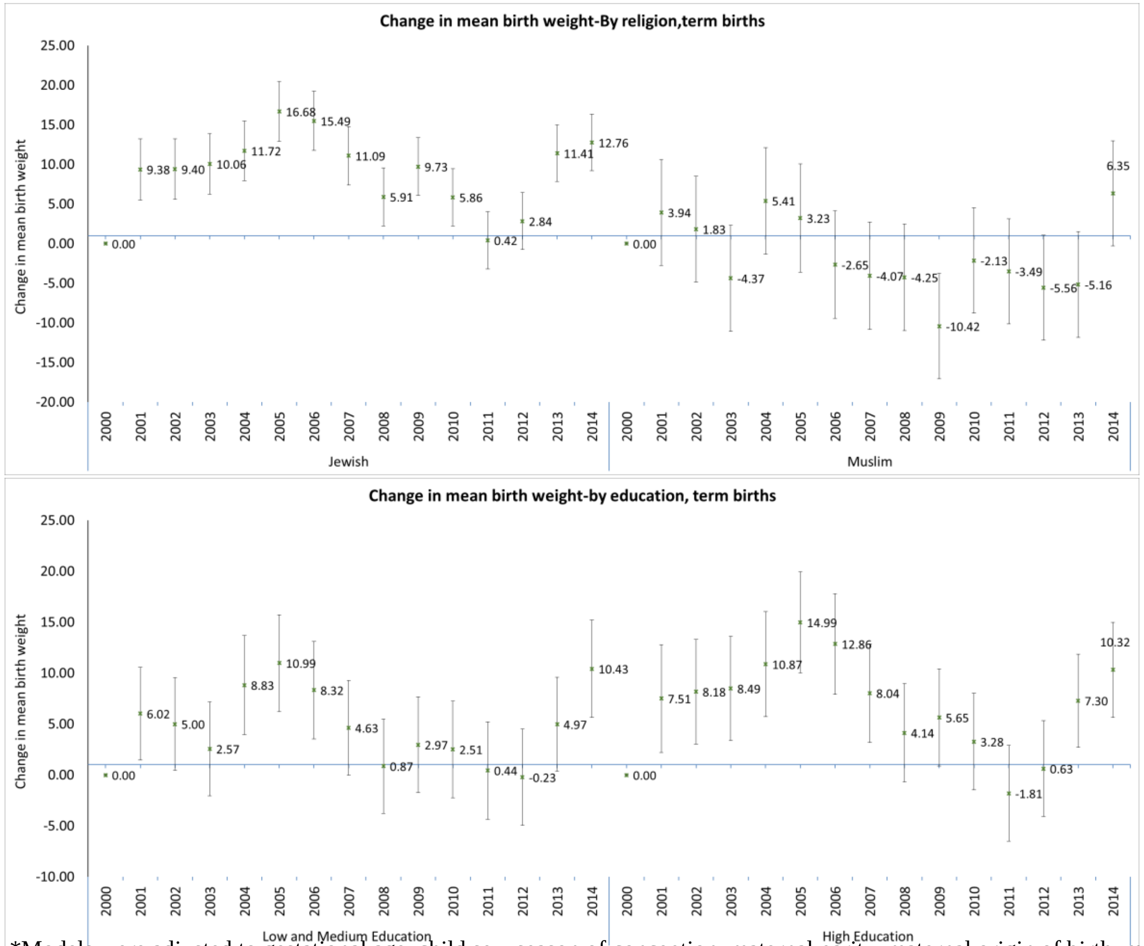

*Models were adjusted to gestational age, child sex, season of conception, maternal parity, maternal origin of birth, maternal family status, maternal age, maternal education and child religion. Term births-births at gestational age equal or above 37 weeks.
